# Insecticide resistant *Anopheles gambiae* have enhanced longevity but reduced reproductive fitness and a longer first gonotrophic cycle

**DOI:** 10.1038/s41598-022-12753-w

**Published:** 2022-05-23

**Authors:** Joyce K. Osoro, Maxwell G. Machani, Eric Ochomo, Christine Wanjala, Elizabeth Omukunda, Andrew K. Githeko, Guiyun Yan, Yaw A. Afrane

**Affiliations:** 1 Entomology Section, Centre for Global Health Research, Kenya Medical Research Institute, Kisumu, Kenya; 2 Department of Biological Sciences, Masinde Muliro University of Science and Technology, Kakamega, Kenya; 3 Department of Medical Laboratory Sciences, Masinde Muliro University of Science and Technology, Kakamega, Kenya; 4 Centre for Global Health Research, Kenya Medical Research Institute, Kisumu, Kenya; 5 Program in Public Health, College of Health Sciences, University of California, Irvine, CA 92697 USA; 6 Department of Medical Microbiology, College of Health Sciences, University of Ghana Medical School, University of Ghana, Accra, Ghana

**Keywords:** Entomology, Evolution

## Abstract

Widespread insecticide resistance in African malaria vectors raises concerns over the potential to compromise malaria vector control interventions. Understanding the evolution of resistance mechanisms, and whether the selective disadvantages are large enough to be useful in resistance management or designing suitable control strategies is crucial. This study assessed whether insecticide resistance to pyrethroids has an effect on the gonotrophic cycle and reproductive potential of malaria vector *Anopheles gambiae*. Comparative tests were performed with pyrethroid-resistant and susceptible colonies of *Anopheles gambiae* colonized from the same geographical area, and the reference Kisumu strain was used as a control. Adult females aged 3 days old were given a blood meal and kept separately for individual egg-laying. The number of days taken to lay eggs post-blood-feeding was recorded to determine the length of the gonotrophic cycle. To measure adult longevity and reproduction potential, newly emerged males and females of equal numbers were aspirated into a cage and females allowed to blood feed daily. The number of eggs laid and the surviving mosquitoes were recorded daily to determine fecundity, net reproduction rate, intrinsic growth rate and adult longevity. Overall, the resistant females had a significantly longer (1.8 days) gonotrophic cycle than susceptible females (F_2, 13_ = 9. 836, P < 0.01). The proportion of resistant females that laid eggs was lower 31.30% (94/300) compared to 54% (162/300) in the susceptible colony and 65.7% (197/300) in the Kisumu strain. The mean number of eggs laid per female was significantly lower in the resistant colony (88.02 ± 20) compared to the susceptible colony (104.9 ± .28.8) and the Kisumu strain (97.6 ± 34.8). The adult longevity was significantly higher for resistant (39.7 ± 1.6 days) compared to susceptible (29.9 ± 1.7 days) and the Kisumu strain was (29.6 ± 1.1 days) (F_2,8_ = 45.05, P < 0.0001). Resistant colony exhibited a lower fecundity (4.3 eggs/females/day) and net reproductive rate (2.6 offsprings/female/generation) compared to the susceptible colony (8.6 eggs/female/day; 4.7 offsprings/female/generation respectively) and Kisumu strain (9.7 eggs/female/day; 4.1 offsprings/female/generation respectively). The study suggests high fitness cost on reproductive parameters of pyrethroid-resistant mosquitoes particularly on the duration of gonotrophic cycle, fecundity and net reproductive rate. These fitness costs are likely associated with maintaining both target site and metabolic mechanisms of resistance to pyrethroids. Despite these costs, resistant mosquitoes had longer longevity. These results give insights to understanding the fitness cost of insecticide resistance and thus are critical when predicting the epidemiological impact of insecticide resistance.

## Introduction

Current efforts to control malaria rely heavily on insecticide-based interventions such as large-scale distribution of long-lasting insecticidal nets (LLINs) and indoor residual spraying (IRS)^1^. Pressure placed upon mosquitoes by the rapid scaling-up of vector control interventions and the use of similar chemicals for agricultural activities have been reported to select for phenotypes with increased ability to survive the insecticides used, gradually impacting vector control efforts^2^. A number of studies have demonstrated that the major insecticide resistance mechanisms involved include decreased sensitivity of the target proteins and increasing activity of detoxifying enzymes (metabolic resistance)^3^. Although these mechanisms confer a significant advantage to the bearers in the presence of insecticides, they have also been associated with a series of side effects in the life history traits of the insect population^4^, which include their biting rate, fecundity and survivorship. However, the expression of these side effects associated with insecticide resistance in *Anopheles gambiae* is unclear.

The biting rate of a vector is dependent on the gonotrophic cycle, which is defined as the time interval between blood-feeding and oviposition^5,6^ and is dependent on the search for a blood meal, blood-feeding and digestion, egg maturation and the availability of a suitable oviposition site^6^. This, therefore, dictates the biting frequency of a vector impacting vectorial capacity and transmission dynamics of malaria^6,7^. Survivorship and fecundity are important determinants of the population growth dynamics of mosquitoes^8,9^. Adult survivorship is one of the fundamental factors of vectorial capacity, a change due to fitness cost could have a profound effect on the disease transmission risks and epidemiology of malaria^8^. For instance, a mosquito needs to survive beyond the extrinsic incubation period of the *Plasmodium* parasites to be able to transmit malaria and a longer lifespan guarantees a potential of biting many hosts^7,9^. Fecundity, a trait measured by the number of offspring an individual mosquito can produce, is a major fitness component since it determines the reproductive rate and intrinsic growth rate of a population^5,10^. Reproductive rate is the average number of offspring produced by a female individual in her lifetime, whilst, intrinsic growth rate is defined as the number of progeny born to each female mosquito per unit time^5^. Reproductive rate and intrinsic growth rate impact directly on the population of mosquitoes which is a major component of vectorial capacity of malaria transmission.

Studies have proven that insecticide resistance impacts the physiological processes of mosquito vectors. These biological processes include adult survivorship, fecundity, blood-feeding, male mating success, development of immature stages and also vector competence^11–16^. Most of these studies were generally carried out under optimal laboratory conditions and involved unrelated resistant and susceptible strains which often differ in many other genes as they originate differently. In addition, the use of reference susceptible strains singly, may not give a true comparison of fitness costs as this population has been maintained in the laboratory for decades and therefore may adapt to laboratory conditions. This study explored the fitness costs of insecticide resistance on the gonotrophic cycle, fecundity and adult survivorship under a semi-natural setting using established colonies of pyrethroid-resistant and susceptible *An. gambiae* with the same origin^17^.

## Materials and methods

### Mosquito population used in the study

The population of mosquitoes used in this study consisted of a resistant colony of *An. gambiae* selected through exposure to deltamethrin insecticide (hereafter referred to as a resistant colony) and unselected susceptible colony of *An. gambiae* that was raised in the absence of insecticides over several generations (hereafter referred to as a susceptible colony). Both resistant and susceptible colonies originated from the same mother colony collected from Bungoma, western Kenya^17^.

#### Resistant colony

The colony was resistant to WHO standard diagnostic dose of deltamethrin (0.05%) with a mortality of 23%. According to WHO, insecticide resistance is difined as ability of mosquitoes to survive exposure to a standard dose of insecticide; this ability may be the result of physiological adaptation^18^. The colony showed both metabolic resistance, through increased monooxygenases enzyme activity and *kdr* mutations with allele frequencies of 77% (L1014S) and 23% (L1014F)^17^ respectively (Supplementary Table S1). The 6th generation was used for this study.

#### Susceptible colony

This colony did not undergo selection at any generation but was monitored for resistance after every generation. This colony had lost phenotypic resistance to deltamethrin after 13 generations (Mortality rate 98%). Only Kdr mutation (L1014S) was detected as it was already fixed in the parent population^17^. The 13th generation was used for this study. The generation difference between the resistant and susceptible colony was due to the delayed development in the selected resistant colony^17^.

#### Kisumu reference laboratory strain

The Kisumu reference laboratory strain which has been colonized since 1954^19^ and is free of any detectable insecticide resistance mechanism was used as a control susceptible strain in all bioassays.

These *An. gambiae s.s* mosquitoes that were used in this study were reared in the insectary at KEMRI/CGHR under standard conditions (25 ± 2 °C; 80% ± 4% relative humidity with a 12 h: 12 h light/dark cycle). Larvae were fed on tetramin baby fish food and brewer’s yeast daily and adults maintained on 10% sugar solution. The resistant and the susceptible colonies used were reared in three lineages as replicates.

### Experimental design

#### Determination of the gonotrophic cycle of the experimental mosquitoes

The study was carried out in a semi-field environment dubbed MalariaSphere located at the Centre for Global Health Research, Kenya Medical Research Institute, Kisumu, Kenya. The MalariaSphere is an enclosed environment with all components of a natural *Anopheles* ecosystem. It is a modified greenhouse with screen walls which contains a local house, planted crops and having breeding sites for mosquitoes to simulate the natural ecosystem of the vector and exposed to ambient climate conditions^20^. Usually such experiments are done under artificial insectary conditions which are very different from real field conditions. Newly emerged (1 day old) males and females from the resistant, susceptible and the Kisumu reference strain were put in separate cages to allow time for mating. On the third-day post-emergency, the females were starved for 6 h before being allowed to feed on the arm of a human volunteer for 20 min. Blood-fed females were provided with 10% sucrose solution, to allow the maturation of eggs. After 24 h (1 day) post-blood-feeding, three hundred (300) fully blood-fed females from each of the colonies were transferred to individual oviposition cups and allowed to lay eggs. The eggs laid by each mosquito were counted and recorded and the number of days taken to lay eggs after blood-feeding was also recorded to determine the length of the gonotrophic cycle. The number of mosquitoes that laid eggs and the number of eggs laid were recorded to determine the daily egg-laying rate and the size of egg batches.

#### Adult longevity and fecundity of resistant and susceptible *An. gambiae*

One hundred (100) newly emerged (one day old) females and males from each of the resistant and susceptible colonies and the Kisumu reference strain were placed separately in 30 × 30 × 30 cm metal-framed cages covered with nylon netting. The cages were suspended from the ceiling of the hut, 2 m above the ground with twine smeared with grease to prevent ants from interfering with the experiment. The hut used in this experiment was erected in an enclosed system and resembled a typical African village house in size and design. Mosquitoes in each cage were fed on 10% sucrose solution through a cotton ball. Females in the cages were starved daily for 6 h thereafter allowed to feed on the arm of a human volunteer. An oviposition substrate consisting of a petri dish lined with a filter paper on wet cotton wool was provided for oviposition. The number of eggs laid were picked and counted under a dissecting microscope daily to determine fecundity. Dead male and female mosquitoes were recorded and removed from the cage daily. The experiment was done in three replicates and simultaneously for each of the resistant, susceptible and Kisumu strains.

### Ethical statement

Ethical approval was obtained from the Kenya Medical Research Institute Scientific and Ethical Review Unit (SERU) under approval number SSC 3434. All experimental protocols were approved by the Instutional Ethics committee. Informed consent was obtained from the subject before the implementation of the study, and all experiments and methods carried out in accordance to the relevant guidelines and regulations of SERU.

### Data analysis

The length of the first gonotrophic cycle was calculated as the average number of days taken for a mosquito to oviposit eggs after taking a blood meal for each colony. The proportion of mosquitoes that laid eggs was calculated as the number of mosquitoes that were able to lay eggs per colony divided by the total number of mosquitoes in the experiment per colony. The average age-specific survivorship for the females was calculated for each colony as the average number of days that the mosquitoes lived per colony. Fecundity was calculated as the average number of eggs laid per individual mosquito per day. The net reproductive rate (R_0_) for each of the three colonies was calculated based on the daily survivorship and fecundity schedule. R_0_ is defined as the average number of offspring a female individual in a population will produce in her lifetime and is calculated as *R*0 = ∑ (*l*_x_*m*_x_), where ∑ is the sum of, *l*_x_ is the age-specific survivorship, and *m*_x_ is the age-specific fecundity per mosquito. Intrinsic per-capita growth Rate (*r*), defined as the number of progeny born to each female mosquito per unit of time, was calculated as *r* = *Ln* (*R*_0_)*/G* where *G* = ∑*l*_x_*m*_x_*x/ ∑l*_x_*m*_x_*,* and *x* is mosquito age^5,21^. Analysis of variance (ANOVA) was used to determine the effect of insecticide resistance on the gonotrophic cycle, fecundity, net reproductive rate and per-capita intrinsic growth rate of the resistant, susceptible and Kisumu strain mosquitoes. Tukey (HSD) was used to test the significance of the difference in the reproductive parameters among the resistant, susceptible and Kisumu strain *An. gambiae.*

## Results

### Duration of gonotrophic cycle

The average duration of the gonotrophic cycle measured from the time of blood-feeding to egg-laying for the resistant females was longer (6.1 ± 0.8 days) than the susceptible females (4.3 ± 1.1 days) and 3.7 ± 0.2 days for the Kisumu strain (F_2,13_ = 9.836, P < 0.003). The average duration of the gonotrophic cycle for the resistant females was significantly longer by 1.8 days than the susceptible colony (F_2, 13_ = 9. 836, P < 0.01; Table 1). Although the duration of the gonotrophic cycle for susceptible females was higher compared to the Kisumu strain, this was not statistically significant (F_2, 13_ = 9. 836, P = 0.570). The proportion of resistant females that successfully laid eggs was lower 31.30% (94/300) compared to 54% (162/300) susceptible colony and 65.7% (197/300) Kisumu strain. The number of resistant females that laid eggs was significantly lower than the susceptible females (F_2,11_ = 0.461, P < 0.05). The average number of eggs laid by an individual mosquito in the resistant colony was lower (88.02 ± 20) compared to the susceptible colony (104.9 ± 0.28.8) and the Kisumu strain (97.6 ± 34.8). The fecundity of the resistant colony was significantly lower than the susceptible colony by 16 eggs per mosquito (F_2,449_ = 6.786, P < 0.001). Although the number of eggs laid per individual mosquito in the Kisumu colony was higher than the resistant colony, this was not statistically significant (P = 0.64).

**Table 1:** Mean duration of gonotrophic cyle and fecundity of resistant and susceptible colonies of *An.gambiae.*

Colony/strain	N	No. laid (%)	Cycle length (days)* Mean ± SD	Fecundity* Mean ± SD
Resistant	300	94 (31.3)	6.1 ± 0.0.8^a^	88.02 ± 20^a^
Susceptible	300	161 (53.6)	4.3 ± 1.1^b^	104 ± 28^b^
Kisumu	300	197 (65.6)	3.7 ± 0.2^b^	97 ± 34^a^

The Gonotrophic cycle is the number of days from blood feeding to egg-laying.

Fecundity is measured in the number of eggs laid per female.

*Values representing the percentage of mosquitoes that laid eggs per colony, mean and standard deviations of gonotrophic cycle length in days and fecundity. The same superscript letters indicate no significant difference.

### Survivorship and fecundity of resistant and susceptible *Anopheles gambiae*

**Figure 1:**
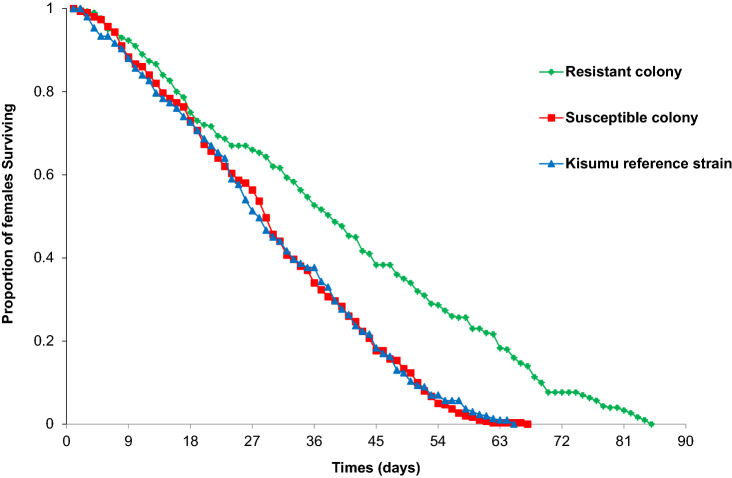
Comparison of survivorship among the resistant, susceptible and Kisumu strain *An. gambiae* females.

The average longevity for the resistant colony was 39.7 ± 1.6 days while the susceptible colony was 29.9 ± 1.7 days and the Kisumu strain was 29.6 ± 1.1 days (F_2,8_ = 45.05, P < 0.0001; Table 2). The average longevity recorded by the resistant colony was significantly longer (by 10 days) than the susceptible colony and Kisumu strain mosquitoes (F_2,8_ = 45.05, P < 0.05). Resistant females survived slightly longer (85 days), with a median longevity of 43 days than the susceptible colony that lived for 67 days with a median longevity of 34 days and the Kisumu strain that survived for 65 days having median longevity of 33 days (Fig. 1).

**Table 2:** Comparison of adult longevity, fecundity, net reproductive rate and intrinsic growth rate among the resistant, susceptible and Kisumu colonies.

Colony/strain	Mean ± SD			
	Longivity (days)	R_0_	*r*	Fecundity
Resistant	39.7 ± 1.6^a^	2.6 ± 0.3^a^	0.215 ± 0.007^a^	4.31 ± 0.9^a^
Susceptible	29.9 ± 1.7^b^	4.7 ± 0.6^b^	0. 285 ± 0.010^b^	8.6 ± 1.0^b^
Kisumu	29.6 ± 1.1^b^	4.1 ± 0.2^b^	0.241 ± 0.013^b^	9.7 ± 0.1^b^

Fecundity is measured in number of female progeny per female per day.

*R*0—is the mean net replacement rate (number of offspring per female per generation).

*r—*is intrinsic per-capita growth rate (number of offspring per female per day).

*Mean and standard deviations of fecundity, net reproductive rate intrinsic per-capita growth rate and survival time. The same superscript letters indicate no significant difference.

The average fecundity of the 3 replicates of resistant females placed in different cages was lower (4.3 eggs per female per day) than susceptible females (8.6 eggs per females per day) and Kisumu strain (9.7 eggs per female per day) (F_2, 9_ = 38.16, P < 0.0004). The average fecundity for resistant females was significantly lower by 4.3 eggs per day than the susceptible females (F_2, 9_ = 38.16, P < 0.001; Table 2). The fecundity was slightly higher in the Kisumu reference strain than the susceptible colony, but the difference was not statistically significant (F_2, 9_ = 38.16, P > 0.05).

### Reproductive fitness of resistant and susceptible mosquitoes

The net reproductive rate calculated for resistant females was lower (2.6 offspring/female/generation) compared to susceptible females (4.7 offspring/female/generation) and the Kisumu strain (4.1 offspring/female/generation, Table 2). Susceptible females exhibited a twofold increase in net reproductive rate over the resistant females (F_2, 9_ = 20.24, P < 0.002), indicating low reproduction for mosquitoes expressing physiological resistance. There was no significant difference in net reproductive rate between the susceptible colony and the Kisumu reference strain (F_2, 9_ = 20.24, P > 0.05).

Similarly, resistant females exhibited a lower intrinsic growth rate (0.215 females /day) compared to susceptible females (0.285) and Kisumu strain (0.241). The intrinsic growth rate was significantly higher for susceptible females compared to resistant females (F_2, 9_ = 41.98, P < 0.0002; Table 2) suggesting that susceptible females will have a major competitive advantage over mosquitoes carrying resistant genes in the absence of insecticide selective pressure.

## Discussion

The emergence and spread of insecticide resistance in malaria vectors of sub-Saharan Africa raise concerns over the control of the disease^22^. Despite the observed resistance, little is known about how insecticide resistance influences the biting frequency, survivorship and reproductive fitness of malaria vectors. Therefore, understanding the effects and mechanisms of insecticide resistance on vector adaptation capacities is crucial to the conservation of susceptibility and the development of more effective resistance management strategies. In this study, we evaluated reproductive fitness of two progenies of *An. gambiae* with the same origin but different insecticide resistance profiles when exposed to pyrethroid insecticides. Overall, results showed that the resistant mosquitoes have a fitness disadvantage on reproductive parameters in comparison to the susceptible strain, suggesting the possible accumulation of deleterious effects of insecticide resistance. However, the survivorship of the resistant mosquitoes was significantly longer compared to the susceptible mosquitoes.

The determination of the gonotrophic cycle, which is a measure of biting frequency is an important parameter in estimating the opportunities for acquisition and transmission of parasites^23^. This study observed a longer first gonotrophic cycle in resistant females compared to their susceptible counterparts suggesting a possible lower human biting rate which could result in lower malaria transmission. These findings corroborate with Mebrahtu, et al.^24^ who observed a delay in the duration of laying eggs for resistant *Aedes aegypti* compared to the susceptible females. The relatively longer gonotrophic period observed in the resistant females possibly could be due to a lower blood digestion rate that may result from the altered physiology of the female mosquito^25^. Previous studies on *Aedes aegypti* have documented the functions of insulin-like peptides and ovary ecdysteroidogenic hormone released from brain neurosecretory cells in the activation of blood digestion^26^. This implies that any changes that directly interfere with the central nervous system may slow or inhibit the release of the hormones required to stimulate the blood digestion process hence inhibiting the process of egg maturation. Although this study did not measure the blood meal size in the resistant and susceptible mosquitoes, some studies have observed a significant reduction of the relative amount of ingested blood in resistant mosquitoes compared to susceptible counterparts^16,24,27,28^. The longer gonotrophic cycle observed in the resistant mosquitoes may suggest reduced biting frequencies and potentially less transmission of malaria parasites in nature, when compared to the susceptible counterparts. However, such mosquitoes with an elongated gonotrophic cycle may have multiple blood meals before laying eggs, which could increase the chances of picking up the malaria parasite and having a high potential for transmitting the disease^6,29–31^. The tendency to take multiple blood meals to complete the egg maturation process has been observed in *Anopheles pseudopunctipennis*^32^ and *Aedes albopictus* ^33^.

The resistant mosquitoes were observed to live longer than susceptible females suggesting that these females could have a longer infective lifespan which may have implications for malaria transmission. The increase in longevity observed in the resistant colony could be a result of the extended larval stage^34^, allowing for a greater accumulation of nutritional resources partially compensating for the losses associated with maintaining the resistance mechanism^35^. Studies with the same colony of mosquitoes showed an extended larval development time of the resistant mosquitoes compared to the susceptible ones^34^. This trait may be critical in promoting the maintenance of resistant individuals in the field, thereby hindering the effectiveness of insecticide resistance management actions. This finding agrees with previous reports of resistant *An. funestus*^14^ that had resistant alleles living longer compared to those with susceptible alleles. On the contrary, some studies have reported a shorter adult lifespan in resistant females such as pyrethroid-resistant *An. coluzzii*^16^ and *Aedes aegypti* with kdr mutations^27^ which were associated with decreased longevity than their susceptible counterparts. In the field scenario, longer survival of resistant mosquitoes will favour the completion of sporogonic cycle of malaria parasites, increased biting of people and potentially transmit disease more than susceptible mosquitoes.

A reduction in the number of egg-laying females was observed in the resistant colony. The number of eggs laid per individual resistant female was lower than in the susceptible colony implying reduced fecundity and egg-laying ability. The variation in fecundity observed may suggest that the nutrients obtained during the blood meal were used for maintaining other processes linked to the survival of resistant females instead of egg production. These findings corroborate with Mebrahtu*, *et al*.*^24^ and Sy*, *et al*.*^28^ who observed a reduction in the number of eggs laid by insecticide-resistant strains of *Ae. aegypti* and *An coluzzii* respectively*.* The reduced ability of resistant mosquitoes to lay eggs could also be attributed to lower insemination rates in insecticide-resistant mosquitoes as reported in other studies^24^, though this trait was not evaluated in this study. The long gonotrophic cycle would also make the resistant mosquitoes take blood meals less often which reduces the egg-laying frequency hence explaining the reduced fecundity of the resistant colony. Although the study reported reduced egg laying ability between the populations, the egg size and viability of the eggs laid was not evaluated, however, some studies have reported reduced viability of eggs laid by deltametrin resistant *Aedes aegypti* when compared to susceptible ones^36^.

The resistant strain exhibited a twofold lower net reproductive rate (*Ro*), which corresponds to the number of females generated from each original female compared to the susceptible strain. Likewise, the intrinsic growth rate was lower in the resistant strain than that observed in the susceptible and Kisumu strain. The loss in the reproductive potential of resistant mosquitoes could be partly attributed to the high levels of resistance and the presence of metabolic resistance mediating the process^17^. The findings on the reduced reproductive fitness as a result of insecticide resistance agree with other studies^16,37^ on *An. funestus* and *Ae. Aegypti* resistant to pyrethroids. In nature, the reduced reproductive fitness in mosquitoes carrying resistant genes could result in a decrease in their progeny limiting malaria transmission.

A limitation of this experiment was the generation difference between the resistant and susceptible populations arising due to delayed development in the selected resistant colony and the loss of resistance in the unselected population. If substantial laboratory acclimation occured then this study would be unable to detect it with these comparisons and difference in acclimation may explain part of the observed difference in longevity and reproductive fitness.

## Conclusion

The study findings show high fitness costs on the reproductive parameters of pyrethroid-resistant mosquitoes particularly on the duration of gonotrophic cycle, fecundity, net reproductive rate and growth intrinsic rates. However, the increased longevity observed in resistant mosquitoes represents a serious threat for disease control, as increased longevity of pyrethroid resistant mosquitoes could lead to an increased level of malaria transmission in regions with high insecticide resistance. The fitness costs observed are likely associated with maintaining both target site and metabolic mechanisms of resistance to pyrethroids. This is critical to determining the extent to which insecticide resistance interacts with mosquito reproduction potential.

## Supplementary Information

Supplementary Table S1.

## Data Availability

The dataset supporting the conclusions of this article is included within the article.
