# Supplementary material for: Insecticide resistant Anopheles gambiae have enhanced longevity but reduced reproductive fitness and a longer first gonotrophic cycle
Source: Sci Rep. 2022 May 23;12:8646. doi: 10.1038/s41598-022-12753-w (PMC9126871; doi:10.1038/s41598-022-12753-w)
Supplement: Supplementary file 1 — Supplementary Table S1. [file 41598_2022_12753_MOESM1_ESM.docx]

**Insecticide resistant *Anopheles gambiae* have enhanced longevity but reduced reproductive fitness and a longer first gonotrophic cycles**

Joyce K. Osoro^1,3^ Maxwell G. Machani^1^, Eric Ochomo^1^, Christine Wanjala^3^, Elizabeth Omukunda^2^, Andrew K. Githeko^4^, Guiyun Yan^5^, and Yaw A. Afrane^6*^

1. Entomology Section, Centre for Global Health Research, Kenya Medical Research Institute, Kisumu, Kenya
2. Department of Biological Sciences, Masinde Muliro University of Science and Technology.
3. Department of Medical Laboratory Sciences, Masinde Muliro University of Science and Technology
4. Centre for Global Health Research, Kenya Medical Research Institute, Kisumu, Kenya
5. Program in Public Health, College of Health Sciences, University of California, Irvine, CA 92697, USA
6. Department of Medical Microbiology, University of Ghana Medical School, College of Health Sciences, University of Ghana, Ghana.

|  |  |  | *Vgsc* | | |
| --- | --- | --- | --- | --- | --- |
|  |  |  | Locus 1014 | | |
| Population | Generation | Mortality (%) | L1014 | L1014S | L1014F |
| Parent population |  | 42 | 0.03 | 0.88 | 0.09 |
| Resistant strain | F6 | 23 | 0 | 0.77 | 0.23 |
| Susceptible strain | F13 | 98 | 0.02 | 0.98 | 0 |

**Supplementary Table 1**. Phenotypic resistance and Kdr Allele frequency of the Parent, Resistant and Susceptible strains of *An. gambiae*
